# The Virginia Medical and Surgical Journal

**Published:** 1853-07

**Authors:** 


*The Virginia Medical and Surgical Journal.* Edited by Geo. A. Otis, M.D., & Howard L. Thomas, M.D.—*Amicus Socrates, Amicus Plato, Sed Magis, Amica Veritas.* Richmond, Va. Published monthly at five dollars a year.

The numbers of this Journal already received, give evidence of industry and ability on the part of its editors.

In their introductory they say: "Ours is a catholic work, the organ of no sect or party; its interests are those of the whole profession; it owes allegiance or obligation to none. With the inflexible resolution to preserve this character, we look to the future with sure faith, and even with the long and dreary history of medical journals before our eye, we cannot believe that we propose another twelve months' voyage to obscurity and the chaos of failures."

It is with physicians as with the people, the greater the number of medical publications, the more they read on those subjects demanding their attention, and the better instructed they become in the principles and practice of their profession. In the families of our farmers, mechanics, and merchants, we seldom see less than two or three periodicals and newspapers. They supply food for the mind, stimulus to thought. It is the same in the professions; the physician who is accustomed to read several medical journals, will, (other qualifications being equal) be a better practitioner, and receive from the profession and the public, more respect than he who reads but one or none at all. We want more light in medicine; we need a greater dissemination of medical truth, and a fuller record of medical facts. Medical periodicals are the means through which this is to be accomplished; but, in order that they may fulfill their mission, they require the aid and the support of the profession.

We believe with some of our eastern friends, that it is a sin to send to medical men, year after year, a valuable scientific journal, without any remuneration, either pecuniary or othewise. Men, who do not value a work sufficiently to pay for it, very seldom read it. We, ourselves, have seen in the offices of physicians, the pages of valuable eastern journals, prostituted to the inglorious purpose of wrapping up calomel and jalap. Just think of it, Messrs. Editors of the Virginia Medical Journal; your articles, on which you have bestowed so much care, and of which you have reason to be a little proud, to be cut up into little square bits, and nicely folded, enclosing a dainty morsel of quinine and carb. of iron, just as if your wisdom and knowledge, by mere contact, could impart a potency to the bitter potion.

But to be serious, we hail the appearance of every new medical journal as a co-laborer in the great field of medical science, as a new beacon for the dissemination of medical light, as a new store house for medical facts. As in literature and politics, the more men read, the greater the demand for literary and political publications; so in the professions, and especially in that of Medicine, the more familiar men become with the condition of our art and science, the greater the demand for our periodicals—the true exponents of our professional progress. J.

